# Middle Georgia Medical Society

**Published:** 1872-03

**Authors:** B. F. Rudisill, R. F. Wright

**Affiliations:** President; Secretary


### MIDDLE GEORGIA MEDICAL SOCIETY.

The first regular meeting of this Society was held in Forsyth, March 6, 1862—Dr. B. F. Rudisill presiding.

The minutes of the preliminary meeting at Barnesville were read and approved.

On motion, the names of the following new members were enrolled: Dr. T. J. Collier, Dr. J. H. Harrison, Dr. B. F. Chambliss, Dr. J. B. Turner.

On motion, the report of the Committee on Constitution and By-Laws was received, and said Committee discharged. The Constitution and By-Laws were then taken up, adopted by articles and as a whole.

On motion, a Committee was appointed to report names of candidates for office. The following names were submitted, and unanimously elected:

For President, Dr. B. F. Rudisill, Forsyth; First Vice-President, Dr. T. R. Kendall, Thomaston; Second Vice-President, Dr. J. O. Hunt, Barnesville; Recording Secretary and Treasurer, Dr. R. F. Wright, Forsyth; Corresponding Secretary, Dr. C. S. Strother, Barnesville.

On motion, the Chairman appointed the following members to report anything new and interesting in connection with the sections specified below:

Anatomy—Drs. Kendall, Cook and Turner; Physiology—Drs. Leseur, Hunt and Shi; Chemistry—Drs. Collier, Harp and Harrison; Materia Medica—Drs. Strother, Gray and Farmer; Surgery—Drs. McDowell, Wright and Rudisill; Obstetrics—Drs. McDowell, Turner and Shi; Practical Medicine—Drs. Strother, Perdue and Kendall; Medical Ethics—Drs. Leseur, Gray and Perdue.

*Resolved*, That a communication expressive of sympathy in affliction, and of regret on account of absence, be forwarded to Dr. Leseur, of Culloden. Also, that he be requested to deliver his promised address at the next regular meeting of the Society, in May.

*Resolved*, That in case of Dr. Leseur's absence, Dr. McDowell be requested to address the Society, on any medical subject, at its next regular meeting.

*Resolved*, That the next regular meeting of the Society shall be held in Thomaston, on the third Wednesday in May.

Drs. S. H. Gray and T. R. Kendall were elected delegates to the Georgia Medical Association.

*Resolved*, That one hundred copies of the Constitution and By-Laws of this Society be published in pamphlet form, and distributed to the members and officers thereof.

*Resolved*, That the Committee on Fee-Bill postpone their report indefinitely.

*Resolved*, That the proceedings of this meeting be published in the ATLANTA MEDICAL AND SURGICAL JOURNAL.

B. F. RUDISILL, M.D., President.
R. F. WRIGHT, M.D., Secretary.

